# Concepts of association between cancer and ionising radiation: accounting for specific biological mechanisms

**DOI:** 10.1007/s00411-022-01012-1

**Published:** 2023-01-12

**Authors:** Markus Eidemüller, Janine Becker, Jan Christian Kaiser, Alexander Ulanowski, A. Iulian Apostoaei, F. Owen Hoffman

**Affiliations:** 1 Institute of Radiation Medicine, Helmholtz Zentrum München, Ingolstädter Landstraße 1, 85764 Neuherberg, Germany; 2 International Atomic Energy Agency, IAEA Laboratories, Friedensstraße 1, 2444 Seibersdorf, Austria; 3 Oak Ridge Center for Risk Analysis (ORRISK, Inc), 102 Donner Drive, Oak Ridge, TN 37830 USA

**Keywords:** Radiation cancer risk, Assigned share, Probability of association, Carcinogenesis, Two-stage clonal expansion model

## Abstract

The probability that an observed cancer was caused by radiation exposure is usually estimated using cancer rates and risk models from radioepidemiological cohorts and is called assigned share (AS). This definition implicitly assumes that an ongoing carcinogenic process is unaffected by the studied radiation exposure. However, there is strong evidence that radiation can also accelerate an existing clonal development towards cancer. In this work, we define different association measures that an observed cancer was newly induced, accelerated, or retarded. The measures were quantified exemplarily by Monte Carlo simulations that track the development of individual cells. Three biologically based two-stage clonal expansion (TSCE) models were applied. In the first model, radiation initiates cancer development, while in the other two, radiation has a promoting effect, i.e. radiation accelerates the clonal expansion of pre-cancerous cells. The parameters of the TSCE models were derived from breast cancer data from the atomic bomb survivors of Hiroshima and Nagasaki. For exposure at age 30, all three models resulted in similar estimates of AS at age 60. For the initiation model, estimates of association were nearly identical to AS. However, for the promotion models, the cancerous clonal development was frequently accelerated towards younger ages, resulting in associations substantially higher than AS. This work shows that the association between a given cancer and exposure in an affected person depends on the underlying biological mechanism and can be substantially larger than the AS derived from classic radioepidemiology.

## Introduction

Exposure to ionising radiation increases the risk of developing cancer later in life. For a person who is diagnosed with cancer after radiation exposure, it is usually assumed that the observed cancer either occurred spontaneously or was caused by the exposure. Recent advances in understanding of the biology of radiation-associated carcinogenesis make it clear that the cancer development cannot be simply separated into a spontaneous part, i.e. without any influence from the studied radiation, and an exclusive part due to the studied radiation. It is possible that radiation exposure accelerates an already existing clonal development towards cancer, and thus with exposure, an otherwise spontaneous cancer will be diagnosed at an earlier age. Furthermore, cancer can be retarded after exposure due to the stochasticity of cellular processes leading to carcinogenesis.

In a recent report on biological mechanisms, UNSCEAR (2021) has identified radiation-induced accelerated growth of pre-cancerous clones (promotion) as carcinogenic mechanism. It was demonstrated in the oesophagus of mice that even low doses (50 mGy) of ionising radiation can lead to the preferential expansion of p53-mutant cells and clones (Fernandez-Antoran et al. 2019). It was shown that this growth acceleration was driven by changes in the oxidative environment of the cells.

Our knowledge on mutational signatures in cancer is rapidly evolving. Modern next-generation sequencing methods allow generation of extensive whole exome and whole genome datasets and to establish a repertoire of mutational processes contributing to the development of human cancer (Alexandrov and Stratton 2014; Alexandrov et al. 2020). Radiation-specific signatures have been suggested in recent years (Behjati et al. 2016; Rose Li et al. 2020). A novel genetic radiation marker has been proposed which is based on the occurrence of radiation-induced gene fusions versus spontaneous point mutations (Morton et al. 2021). It is expected that the possibilities to elucidate the role of radiation in cancer development will dramatically increase in the coming years and decades.

Cancer induction and promotion of pre-neoplastic clones by ionising radiation are stochastic processes. In the absence of specific biomarkers that indicate whether a given cancer has been initiated or accelerated by radiation exposure, the relationship between radiation and an observed cancer can, therefore, only be expressed by probabilities. With the new sequencing methods, in the future, we might be able to derive relations between specific cancer types and preceding exposures. If the tumour was associated with the studied radiation, we could be faced with questions such as: Was the exposure a necessary requirement for tumour development? Was it acting on an early or late stage of development? Did radiation accelerate an already existing process?

In some countries, including the US and Germany, claims for compensation for cancer after occupational exposure are judged based on the assigned share (AS), also called probability of causation (Kocher et al. 2008; Ulanowski et al. 2020). The AS is calculated from an assessment of radiation-induced and spontaneous baseline cancer rates in radioepidemiological cohorts, where baseline refers to an unexposed population. The definition of AS relies on the studied radiation causing additional cases of cancer that would not occur in the absence of exposure. As was pointed out by Greenland and collaborators in a series of publications, (e.g. Beyea and Greenland 1999; Greenland 1999; Greenland and Robins 2000), radiation could affect ongoing carcinogenic processes and accelerate an already existing clonal development towards cancer. In Beyea and Greenland (1999), the authors compared two models with different biological radiation effects. Both models predicted 100 spontaneous cancer cases and 10 additional radiation-induced excess cases. In the first model, only the 10 excess cases were induced by radiation, and the 100 spontaneous cases were unaffected by the exposure. In the second model, also all 100 spontaneous cases were accelerated towards younger ages resulting in loss of cancer-free life, and therefore all observed cases were associated to radiation. Although this thought experiment illustrates the issues related to attributing a cancer to radiation exposure, these theoretical considerations do not have support from experimental or epidemiological data.

The biologically based two-stage clonal expansion (TSCE) model describes the stochastic development towards cancer via an initiation of healthy stem cells to become pre-cancerous, a phase of clonal expansion of the pre-cancerous cells with cell division and differentiation/inactivation, and a transformation step creating a malignant cell. The parameters of such model can be assessed by analyses of radioepidemiological cohorts (Rühm et al. 2017). Therefore, the TSCE model provides a framework that includes major biological mechanisms necessary to study associations between cancer and exposure. The model has been shown to be consistent with age patterns of observed cancer risks and to describe well large radioepidemiological data sets (Meza et al. 2008; Luebeck et al. 2013; Rühm et al. 2017; NCRP 2020; UNSCEAR 2021).

The aim of the current work is to define and study concepts of association between cancer and radiation with different biologically plausible models supported by epidemiological data, and to investigate the consequences of radiation-induced cancer acceleration. Here, values of parameters of three TSCE models with different radiation mechanisms were derived so that the models reproduce the trends of breast cancer data from the atomic bomb survivors of Hiroshima and Nagasaki. Using these models, Monte Carlo simulations were performed to quantify different associations between cancer and exposure for a specific scenario.

## Methods

### Phenomenological and mechanistic points of view

**Fig. 1:**
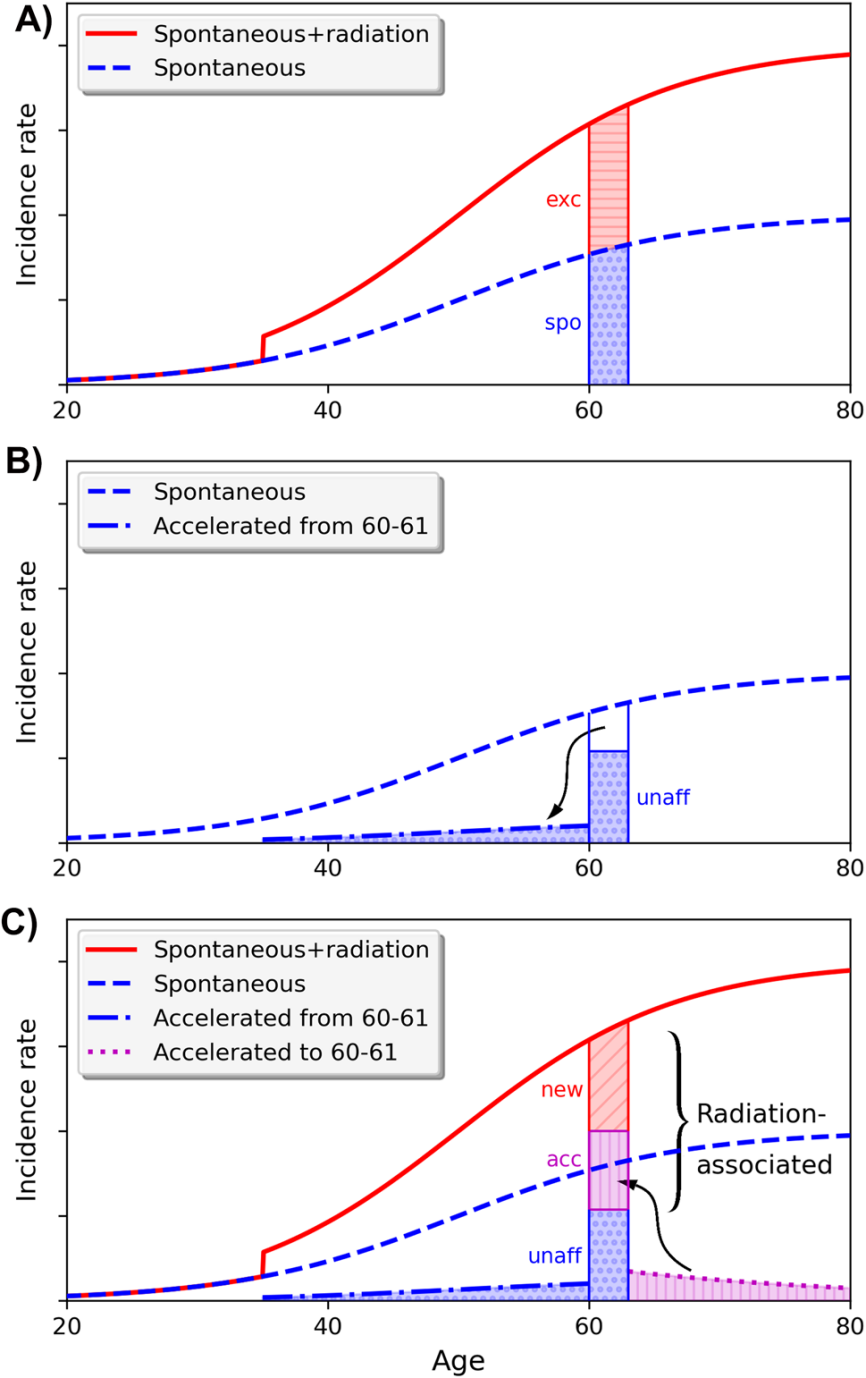
Schematic representation of cancer initiation and acceleration. **A** Radiation exposure at age 30 leads to a doubling of the spontaneous incidence starting at age 35. Within the 1-year age interval 60–61 (shown enlarged), the same number of spontaneous (spo) and radiation-induced excess (exc) cases are expected. **B** For a number of people with a spontaneous cancer between 60 and 61 in the absence of radiation, the exposure accelerates these cancers towards lower ages, others remain unaffected (unaff). **C** For a number of people with a spontaneous cancer in the age interval 61–80 in the absence of radiation, the exposure accelerates (acc) these cancers to age 60–61. The rest of the cases are newly radiation induced (new) and the persons would not get a spontaneous cancer until age 80. For the exposed persons with a cancer in the age interval 60–61, the fraction of cancers that is associated with radiation is larger than 50%. A potential retardation effect (ret) is not shown

To illustrate the underlying concepts of association of cancer and radiation regarding cancer induction and acceleration, and to clarify the terminology, it is helpful to start with a schematic example, shown in Fig. 1.

Let us assume a large cohort in which the cohort members are followed until the age of 80.0 years. All cancer cases are recorded, and in the absence of radiation this would result in a baseline incidence rate ${\lambda }_{0}\left(a\right)$, depending on attained age *a*. Now, all cohort members receive the same radiation exposure at age 30. As a consequence of this exposure, it is assumed that—after a lag time of 5 years—the total incidence rate doubles compared to the baseline incidence rate, so $\lambda =2\cdot {\lambda }_{0}$ at all ages 35 years and above. Within a specified age interval, e.g. within 1 year between 60.0 and 61.0 years, in the following denoted by 60–61, 100 spontaneous baseline cases would have been observed in the absence of exposure, but this number doubles to 200 cases with radiation, as shown in Fig. 1A. This corresponds to an excess relative risk (ERR) of 1 and an assigned share, AS = ERR/(1 + ERR), of 0.5.

We further assume that radiation can be responsible for two biological effects: (i) induction of changes in healthy cells that can put them to a path to cancer (initiation), (ii) acceleration of an already existing progress towards cancer (clonal expansion/promotion of pre-cancerous lesions). In this example, we do not consider potential retardation effects of radiation. Let us investigate the origin of the cancer cases that occur in the age interval 60–61. A fraction of the 100 spontaneous cases (e.g. 70 cases) are not affected in any way by the radiation exposure, so they occur in this age interval without and in the presence of the studied radiation. However, other 30 spontaneous cases are now accelerated by radiation towards earlier ages and are diagnosed before the age of 60. Some of these cases may be accelerated only by a few years, while others by many years (Fig. 1B). At the same time, some cases that spontaneously would have occurred only after age 61, are now accelerated to the time interval 60–61. If the acceleration time was less than 19 years, i.e. the cancers would have spontaneously occurred between 61 and 80, the cancers in the affected persons appear earlier and the persons lose years of cancer-free life (e.g. 50 cases). Furthermore, for the rest of the observed cases (80 cases), the affected persons would not develop a spontaneous cancer until the end of follow-up of 80 years, so the cancers appear as new radiation-induced cases (Fig. 1C). These new cases might either stem from cells that were initiated by radiation, or from clones whose cancer development was accelerated by more than 19 years.

Now, we have a person of the cohort who is diagnosed with cancer within the age interval 60–61. What is the probability that the observed cancer is related to the preceding radiation exposure? Two different points of view can be used to quantify the relationship between radiation and cancer:

*Phenomenological point of view*: from a population/cohort level, 50% of the cancers between 60–61 are caused by radiation, so the assigned share (AS) of excess cases to total cases is AS = 100/200 = 0.5 (Fig. 1A).

*Mechanistic point of view*: for the affected person, as might be indicated by a radiation marker for biological mechanisms, the situation is fundamentally different. The probability that the cancer diagnosed at age 60–61 in this particular person is associated with radiation accounts for both radiation-induced and radiation-accelerated carcinogenesis. The probability of association (PA) is, therefore, given by PA = (80 + 50)/200 = 130/200 = 0.65 (Fig. 1C).

It is important to note that the calculation of PA requires an assessment of the number of cases that are newly induced or accelerated, and therefore depends on the underlying biological model. In both points of view, the spontaneous baseline and total incidence rates of cancer in the cohort are identical. However, from an estimate of the spontaneous and excess cases alone, it is not possible to calculate PA.

In the presence of acceleration, the number of unaffected cases is smaller than the number of spontaneous cases because, after exposure, some of the cancers appear at earlier ages and are moved out of the age interval 60–61. These 30 cases should, therefore, not be explicitly included in the calculation of PA for exposed persons with a cancer in 60–61, but they contribute to the calculation of PA for other age intervals.

Assuming we had a radiation marker from the tumour tissue of every person with cancer between 60–61 that could indicate whether the cancer was unaffected by radiation, accelerated by radiation, or newly induced by radiation, in principle the marker is supposed to show 70 unaffected, 50 accelerated and 80 newly radiation-induced cases. However, a biological marker is indifferent about the end of follow-up. For persons with a spontaneous cancer after the end of follow-up of 80 years, whose cancer was accelerated by more than 19 years, the marker would still indicate an accelerated cancer. Therefore, the marker could show more accelerated cases and less newly induced ones. Nevertheless, the sum of accelerated and newly induced cases as shown by the marker would still be 130 cases.

This was an example using fictitious numbers to illustrate the concept. In the following sections, we present a Monte Carlo simulation with rigorous biologically based models derived from breast cancer data of the Life Span Study (LSS) cohort of the atomic bomb survivors. Different models with radiation effects on initiation and clonal expansion are analysed separately to better understand the influence of the different mechanisms on the probability of association.

### Risk models

In this study, the biologically based two-stage clonal expansion (TSCE) model was used as model framework. This model has been applied in analyses of many radioepidemiological cohorts and includes a mathematical description of the processes of the initiation and promotion of initiated cells. Extensive reviews on the TSCE model and its extensions have been presented by Rühm et al. (2017), NCRP (2020) and UNSCEAR (2021). Therefore, here we describe only the main properties relevant for the current analysis.

**Fig. 2:**
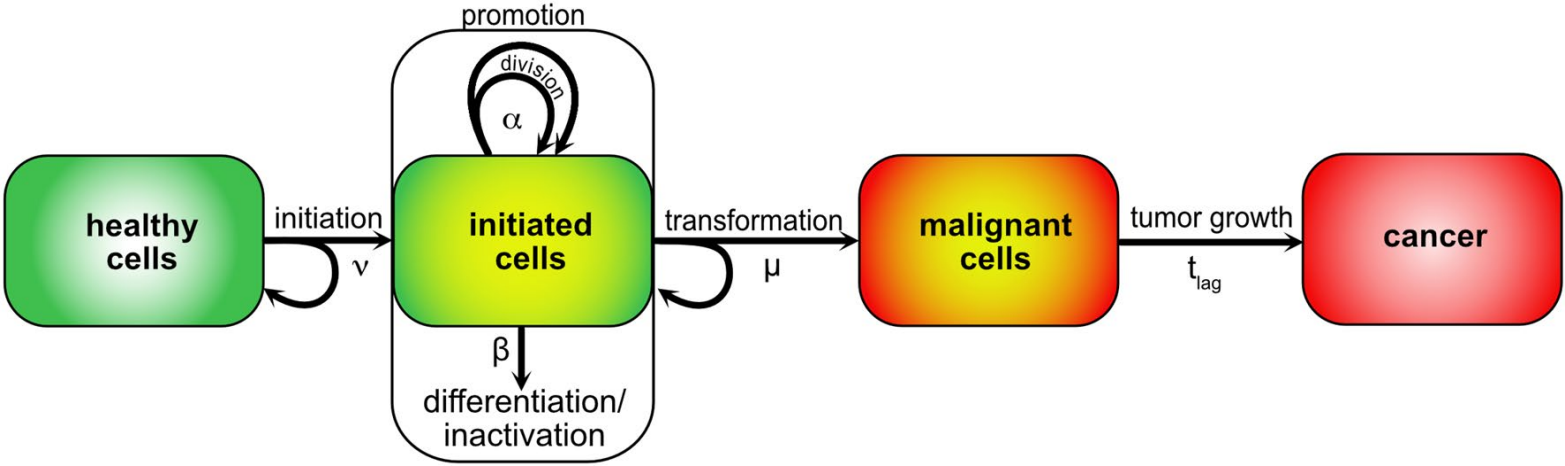
Two-stage clonal expansion (TSCE) model

As shown in Fig. 2, the TSCE model describes the development from healthy cells towards malignant cells and finally cancer. In a first step, healthy stem or progenitor cells are initiated, e.g. by a mutation, giving them a competitive advantage over the neighbouring cells and providing them with the potential to grow into a clone with a larger number of initiated cells. With a pool of $N$ stem cells and an initiation rate per cell of $\nu$, the rate of production of new clones is given by $N\cdot \nu$. In this work, rates are always given in units of events per year. Since the processes are stochastic, the distribution of the number of initiated clones after a certain time interval is given by a Poisson distribution (Kai et al. 1997). Once created, the number of initiated (pre-cancerous) cells in the clones can expand during a phase often called ‘promotion’. The initiated cells can symmetrically divide with rate of $\alpha$, building a pool of potential cancer cells. Furthermore, they can drop from this pool by differentiation or by inactivation (e.g. apoptosis or cell killing). The rate of differentiation/inactivation is represented by the parameter $\beta$. Healthy stem cells in adults are in homeostasis, i.e. symmetric cell division on one side and differentiation and inactivation on the other side are balanced. Initiated cells, however, have a growth advantage with α > β. The dynamics of clonal growth and cancer development are strongly driven by the effective clonal expansion rate $\gamma \approx \alpha -\beta$. Additional mutational events can induce a malignant transition from an initiated to a cancer cell. Such transformation events occur in the model with rate $\mu$ per initiated cell. The probability of a transformation event increases linearly with the number of initiated cells. It is assumed that the malignant cell finally grows into an observable tumour after some lag time ${t}_{lag}$.

The stochastic processes of cancer development occur spontaneously, and the model has been shown to reproduce well the age dependence of many cancer rates. In principle, radiation can change any of the biological parameters. Usually it is assumed that radiation has an instantaneous effect, i.e. the baseline values of parameters change during the exposure and return to their previous values afterwards. However, it is also possible to introduce lifelong effects where the parameters remain modified even after the radiation has ceased. This is a way to effectively explain biological effects such as genomic instability (Jacob et al. 2010; Eidemüller et al. 2015).

In this work, values of TSCE model parameters were derived so that the model reproduces the trends of breast cancer data from the Life Span Study (LSS) of the atomic bomb survivors of Hiroshima and Nagasaki with a follow-up from 1958 to 1998. The cohort included 61,977 women and 1,038 primary breast cancer cases with a person-year weighted mean dose to breast tissue of 86 mGy for the full cohort.

This dataset was analysed by Kaiser et al. (2012) with a large variety of different TSCE models. Here, the aim is to investigate consequences of radiation-induced initiation and promotion; therefore, only a reduced and simplified set of models were used as compared to Kaiser et al. (2012). Three models with different relevant mechanisms were selected. The first model (${M}_{I}$) assumes an instantaneous radiation effect on initiation, i.e. the initiation rate $\nu$ increases during exposure and returns to its spontaneous value afterwards. Such a radiation effect is often found in analyses of radioepidemiological data after exposure to low-LET radiation. The second model (${M}_{P}$) has an instantaneous radiation effect on promotion, i.e. the clonal expansion rate $\gamma$ increases during exposure, but it has no radiation effect on initiation. Such an effect is indicated by many studies both after exposure to low-LET and high-LET radiation. The last model (${M}_{P-ll}$) has only a lifelong radiation effect on promotion so that the clonal expansion rate remains permanently elevated after the exposure. Such a model was found by Kaiser et al. and is compatible with a potential effect of genomic instability on growth rates. The model ${M}_{P-ll}$ serves as comparison to the model ${M}_{P}$ since they share a similar mechanism but have different dose responses and age dependencies. These models correspond to the models ${M}_{1}$, ${M}_{3}$ and ${M}_{4}$ in the reference by Kaiser et al. (2012), and all models fitted the epidemiological data reasonably well.

While Kaiser et al. (2012) used baseline parameters that depend on age, here the models were re-fitted with age-independent baseline parameters. An age dependence of the baseline parameters is irrelevant for the demonstration purposes of the current work, which investigates the effects of different radiation mechanisms. Moreover, such parametrization would make the interpretation of results less transparent. Although included by Kaiser et al., no models with a radiation effect on the transformation towards malignant cells were considered since they were not the focus of the current work, and because, from other studies, there is very little support for an effect of radiation on transformation (Rühm et al. 2017; UNSCEAR 2021).

Table 1 shows the parameters of the three models. For the first two models ${M}_{I}$ and ${M}_{P}$, the instantaneous radiation effect is implemented by a linear dependence of the initiation and clonal expansion rate, respectively, on dose rate $d$. The lifelong radiation effect is implemented by a linear dependence of the clonal expansion rate on total breast dose ${D}_{tot}$, so that the clonal expansion rate remains elevated even when the dose rate returns to zero. Since only three of the four biological baseline parameters can be determined from epidemiological data, an additional assumption is required to fix all four biological parameters (Heidenreich 1996; Rühm et al. 2017). Here, the division rate was set to a monthly cycle, with a value of $\alpha =12$ per year as was used in other studies of breast cancer with biologically based models (Eidemüller et al. 2015). The results on the assigned share and probability of association are independent of this assumption, and this was also checked and confirmed by repeating the simulation with different values of $\alpha$, e.g. with $\alpha =1$ per year. For a fixed value of $\alpha$, an increase of the clonal expansion rate $\gamma$ by radiation translates into a reduction in the value of parameter $\beta$.

**Table 1:** TSCE models and parameter values. The dose rate ${\varvec{d}}$ is given in Gy/year, the total dose ${{\varvec{D}}}_{{\varvec{t}}{\varvec{o}}{\varvec{t}}}$ in Gy

Model	${M}_{I}$	${M}_{P}$	${M}_{P-ll}$
Mechanism	Initiation (instantaneous)	Promotion (instantaneous)	Promotion (lifelong)
Radiation effect	$\nu = \nu_{o} \left( {1 + 52.4 \cdot d} \right)$	$\beta = \beta_{0} - 2.3 \cdot d$	$\beta = \beta_{0} - 0.112\ year^{ - 1} \cdot D_{tot}$
$N\cdot {\nu }_{0}$ [events/year]	0.054	0.177	0.076
${\alpha }_{0}$ [events/year]	12	12	12
${\beta }_{0}$ [events/year]	11.837	11.913	11.867
${\mu }_{0}$ [events/year]	$3.83\cdot {10}^{-6}$	$4.64\cdot {10}^{-6}$	$4.30\cdot {10}^{-6}$

Traditionally, AS is calculated on the basis of phenomenological models, in which the radiation response is modelled by a dependence of the excess relative risk (ERR) on dose. To compare with this approach, we also include an ERR model in our calculation. In the preferred (E4) model of Kaiser et al. (2012) for breast cancer in the LSS, the ERR depends linearly on the total breast dose${D}_{tot}$. It is described by ${\text{ERR}}\left( {D_{tot} , a} \right) = 1.06 \cdot D_{tot} \cdot {\text{exp}}\left( { - 1.92 \cdot \ln \left( {a/70} \right)} \right)$, where $a$ represents the attained age. The full hazard is given by $h\left( {D_{tot} ,a} \right) = h_{0} \left( a \right) \cdot \left( {1 + {\text{ERR}}\left( {D_{tot} ,a} \right)} \right)$, and the baseline hazard is $h_{0} \left( a \right) = {\text{exp}}\left( { - 8.07 - 3.37 \cdot \ln \left( a/70 \right) - 7.29 \cdot \ln^{2} \left( a/70 \right) + 8.87 \cdot \left( {\max \left[ {0,\ln \left( a/51 \right)} \right]} \right)^{2} } \right)$. For the given exposure scenario, the expected number of spontaneous, total and excess cases in the age interval 60–61 can be directly calculated as well as the ERR and AS. Because this model does not explicitly account for biological mechanisms, it is not possible to calculate accelerated or retarded cases and, consequently, it does not allow calculation of association measures.

For the studied exposure scenario, it can be expected that predicted numbers of spontaneous and excess cancer cases differ between models, but that they should be of comparable size. This is confirmed by our simulation study. Nevertheless, the interest is not in the absolute numbers, but in the relative contribution of radiation-associated cases.

### The Monte Carlo simulation

To study the influence of radiation on cancer development and to investigate whether a cancer was newly induced or accelerated by radiation, it is necessary to track the behaviour of cells and clones in each single person, with the process being repeated for all persons. This can be achieved via stochastic simulation using the Monte Carlo method. The creation of clones with initiated cells, their cell division and differentiation/inactivation, and their transformation to malignant cells are simulated as stochastic processes. In a simulation without radiation, the number and size of the clones at each age interval are tracked until the end of the follow-up. In parallel, the changes in the spontaneous development due to radiation are simulated. Radiation can create new clones, or affect the division or differentiation/inactivation of initiated cells in existing clones. If the person develops a cancer before the end of follow-up, it is evaluated whether this cancer was unaffected by radiation, newly induced, accelerated or retarded.

The selected scenario for the analysis was a single exposure lasting 1 year that delivered a total dose to the breast tissue ${D}_{tot}$ of 1 Gy at age 30 with a dose rate $d$ of 1 Gy/year. A fixed time lag ${t}_{lag}$ of 5 years from a transformation event, creating a malignant cell, to an observable cancer was assumed. All cancer cases within the 1 year interval 60–61 years were scored. The follow-up continued until age 80.0. For the LSS, typically an age at exposure of 30 years and an attained age of 70 years are chosen. In our study, instead an age at cancer between 60 and 61 was selected so that it was possible to study cancer acceleration over a longer period of 19 years until age 80. For 1,000,000 identical persons, the cellular development was simulated to achieve sufficient statistical power. To evaluate uncertainties, the simulations were repeated ten times for each of the three TSCE models with different (consecutive) seed numbers for the random number generator. No individual variation between the persons was assumed, so, for all persons, the same LSS-derived baseline and radiation parameters of Table 1 were used. For each person, the possible cancer and its association to radiation were identified as described below. The software code was written in C +  + . Since the implementation of models in the simulation is important for interpretation of the results, the procedure is presented in more detail below.

**Fig. 3:**
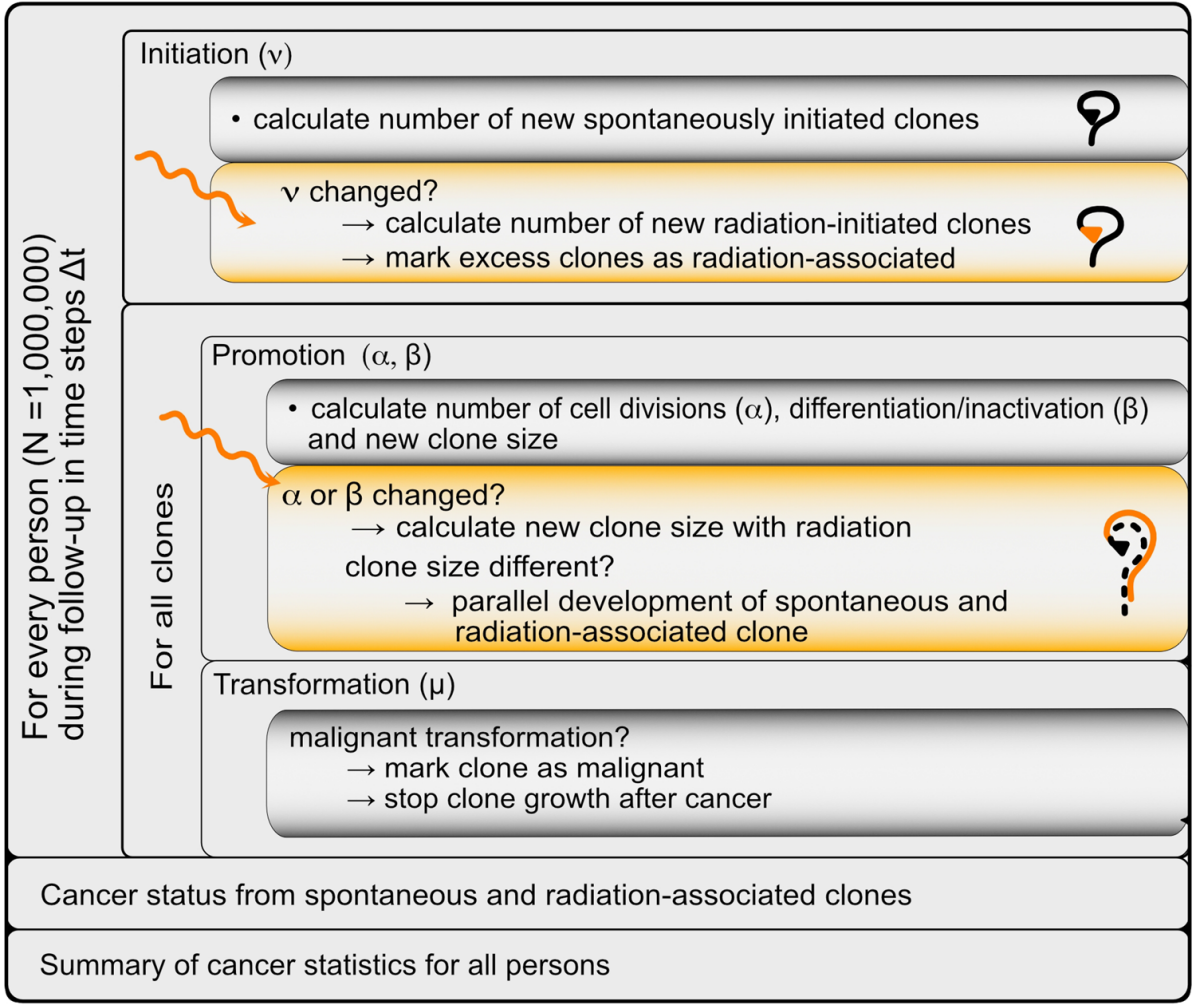
Simulation flow. The curvy lines represent the clone evolution as explained in Fig. 5

The simulation flow is shown in Fig. 3. For each person, the simulation runs from birth until age 80 in time steps of ${\Delta }t$. To achieve a good approximation to the analytical solution of the model, the time steps are chosen such that the product of any rate and ${\Delta }t$ remains below 1/25. Starting at birth without initiated cells, the spontaneous clone evolution is simulated. At a particular time $t$, the person has $n_{c} \left( t \right)$ clones of various sizes. The development of each of these clones is followed separately. To simulate the changes from $t$ to $t + {\Delta }t$, the following calculations are performed: (i) the number of newly created clones, ${\Delta }n_{c}$, is sampled from the Poisson distribution $P\left( {N \cdot \nu \cdot {\Delta }t} \right)$, where $N \cdot \nu \cdot {\Delta }t$ is the mean number of newly initiated cells. Each of these cells forms the origin of a new clone that consists of one initiated cell. (ii) Then the simulation follows all $n_{c} + {\Delta }n_{c}$ clones. Each clone $i$ consists of $m_{i}$ initiated cells that may divide, differentiate or die, or transform into a malignant cell. The probabilities per cell for these events are given by $\alpha \cdot {\Delta }t$, $\beta \cdot {\Delta }t$ and $\mu \cdot {\Delta }t$, respectively. Accordingly, in any one time step, the clone may remain unchanged, grow, become smaller, extinct, or develop a malignant cell that will turn into detectable cancer after $t_{lag}$.

**Fig. 4:**
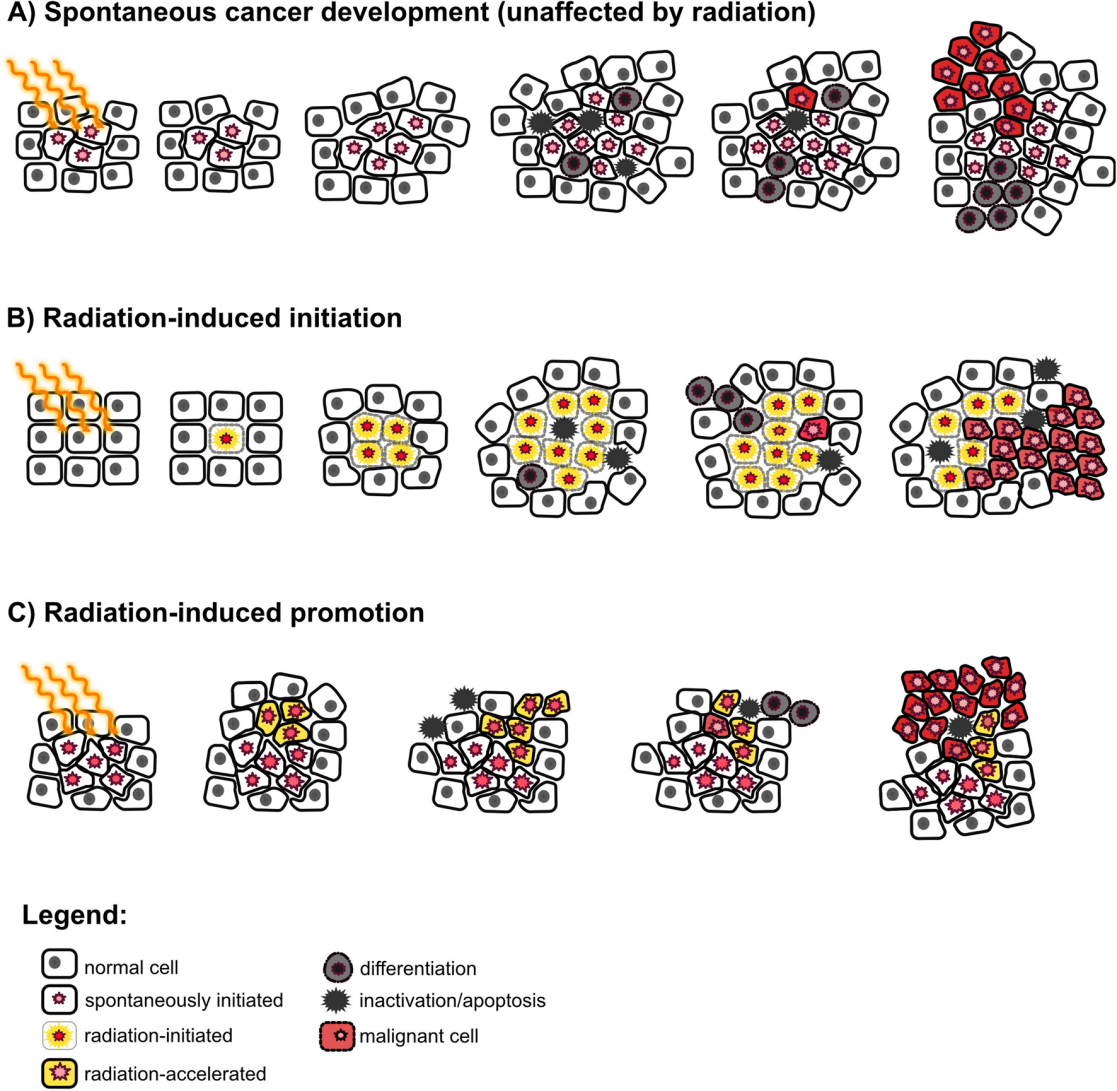
Possible cancer development after radiation exposure. **A** The radiation exposure leaves no imprint on the spontaneous cancer development: tissue contains a clone with initiated cells with a growth advantage. Multiple cycles of cell division and differentiation/inactivation can lead to clonal expansion of the initiated cells. Further mutational changes can create a malignant cell that develops into an observable tumour. **B** Radiation-induced initiation: radiation creates an initiated cell. The initiated cell can expand clonally and develop into a tumour. **C** Radiation-induced promotion: radiation accelerates clonal expansion of an existing clone and may lead to earlier appearance of cancer. In **B** and **C,** the clone is marked as 'radiation-associated' by the simulation

These processes occur spontaneously or may be altered by radiation. During and after radiation exposure, the radiation-induced changes are followed in parallel to the spontaneous development. If radiation increases the parameter υ and, thus, influences the initiation process at a certain time, the number of newly initiated clones is simulated from the total initiation rate for this time step. The difference between these total new clones and the spontaneously created new clones represents radiation-induced excess clones. These clones are labelled as ‘radiation-associated’ in the simulation. If one of these excess clones leads to cancer at some later time, the cancer is classified as associated to radiation. Similarly, if radiation acts on promotion (implemented by a change of β with dose rate during exposure or lifelong with total dose) at some time, the corresponding processes of cell division or differentiation/inactivation are calculated separately with and without the radiation action during the time step. If the size of the clone remains unchanged, it is assumed that radiation has no influence on the future clone development. However, if the size of the clone is changed by radiation, future processes of cell division and differentiation/inactivation are simulated independently of the processes that would take place in the original clone without radiation. The reason is that intercellular signalling processes of clones of distinct sizes are different, and it is, therefore, assumed that after a radiation-induced change of clone size, the processes of cell division and differentiation/inactivation can be regarded as uncorrelated compared to the unexposed clone. Therefore, after the event, the clone is followed by two independent parallel simulations. If a cancer develops from the radiation-modified clone, it is classified as radiation associated, else it is classified as spontaneous cancer. Figure 4 shows graphical presentations of the processes.

In the absence of cancer, the simulation is performed until age 80. If a person develops cancer from a clone that is unaffected by radiation, any clone growth after the age of cancer is stopped. If cancer develops from a radiation-associated clone, any further growth of radiation-associated clones is stopped, and, correspondingly, growth of clones that are related to spontaneous development is stopped after occurrence of spontaneous cancer. Thus, for each person, only the first cancer from spontaneous and the first cancer from radiation-associated development are recorded.

**Fig. 5:**
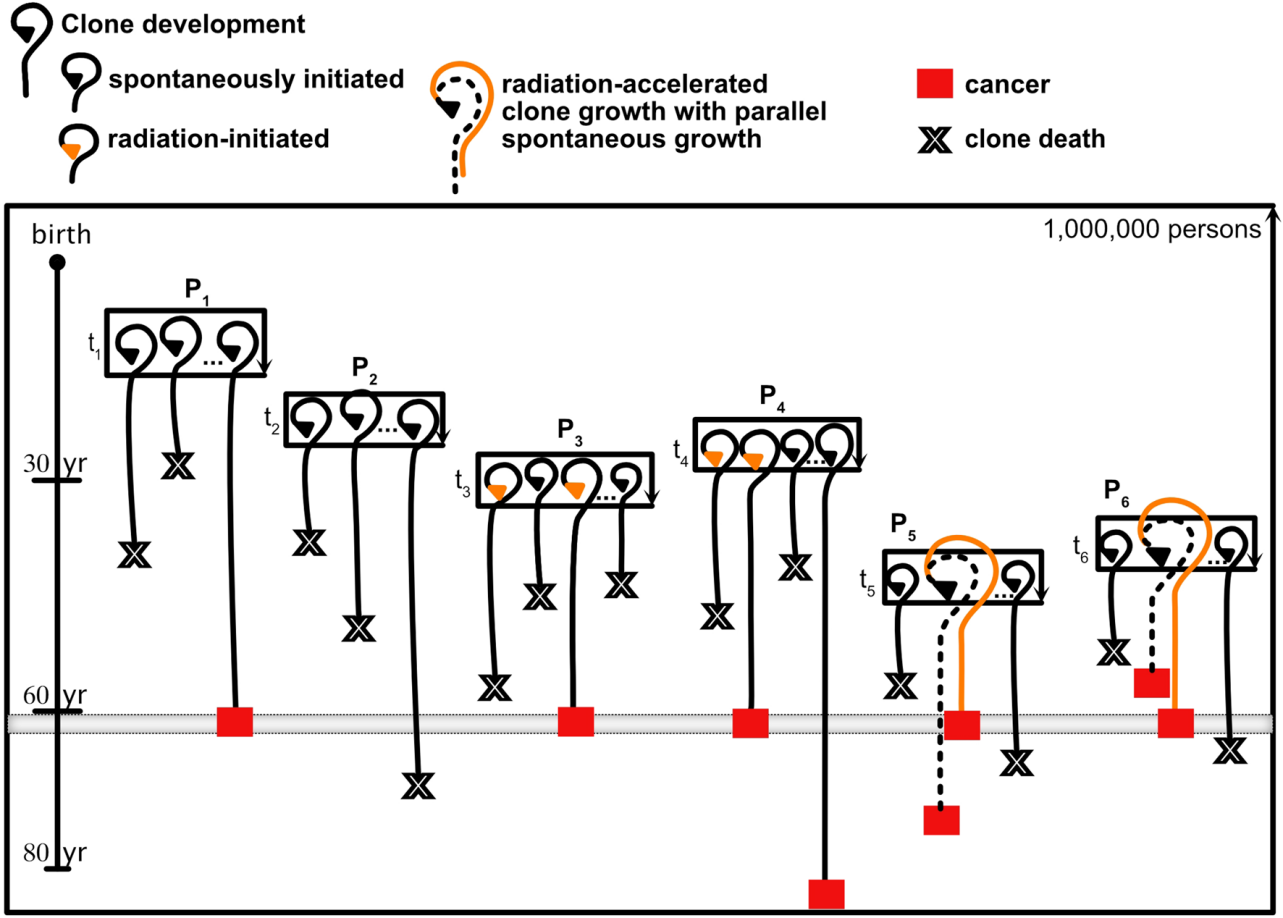
Scoring of cancer cases. Clones of different persons are shown at several time steps. The time evolution of a clone is symbolised by a curvy line and depends on the parameters $\alpha$ and $\beta$. The small triangle indicates the initiation process representing the parameter $\nu$. Person 1 develops a spontaneous cancer in the age interval 60–61, whereas for person 2, all clones die out. Person 3 develops cancer from a radiation-initiated clone and is counted as radiation-associated new cancer case. Person 4 would have developed a spontaneous cancer in absence of radiation, but only after the follow-up time, so it is counted as radiation-associated new cancer case like person 3. Person 5 gets cancer in the age interval 60–61 from a clone with radiation-accelerated growth. Without radiation, the clone would have developed cancer between 61 and 80, so it is a person with radiation-associated accelerated cancer. Person 6 would have developed a spontaneous cancer before age 60, but in the presence of radiation, it is retarded to 60–61, so it is counted as radiation-associated retarded cancer case

Finally, an evaluation over all persons was performed as presented in Fig. 5. For each person, if a cancer developed in the age interval 60–61 from a radiation-associated clone, but no cancer developed spontaneously until the end of follow-up, the cancer was marked as “new radiation-associated cancer”. If also a spontaneous cancer was observed, but in the age interval 61–80, the cancer was marked as “accelerated cancer”. With a radiation action on promotion, also a retardation of cancer development is possible. After a change of clone size by radiation, cell division and differentiation/inactivation are independently simulated for the radiation-associated and for the original clone. Due to the stochasticity of these processes, it can, e.g. happen that the radiation-associated clone becomes smaller than the original one and that the radiation-associated cancer appears later. Such cases were marked as “retarded cancer”. From these quantities, the assigned share and different measures of association were calculated.

The results of the simulation for the cancer rates, ERR and EAR were checked against an analytical solution of the TSCE models, and were found to be in excellent agreement (results not shown). However, the analytical solution does not contain information about individual clone development which was the focus of the current study.

### Measures of association

After the simulation, the assigned share AS for the age interval 60–61 was calculated by the difference between the total and spontaneous cases divided by the total cases. This corresponds to the usual definition of the assigned share based on radioepidemiological cohorts.

Without a retardation effect, as in the simplified example of Fig. 1, the probability of association is defined by the sum of cases of radiation-induced new or accelerated cancers, divided by the total cases. In the presence of retardation, however, there are several quantities of interest to characterise the association between cancer and radiation. These include:

- PA: probability of association, PA = (new + accelerated + retarded)/total: any association between cancer and radiation, includes both harmful (new and accelerated cases) and retardation associations.
- PH: probability of harm, PH = (new + accelerated)/total: harmful association between cancer and radiation.
- PEH: probability of effective harm, PEH = (new + accelerated-retarded)/total: effective harmful association by subtracting the retarded cases from the harmful cases. This reduces the stochastic fluctuations of cell division and differentiation/inactivation, leaving the effective harmful effects of exposure.
- PR: probability of retardation, PR = retarded/total.
- HRR: harm / retardation ratio, HRR = PH/PR = (new + accelerated)/retarded: ratio of harmful to retardation effects.

**Fig. 6:**
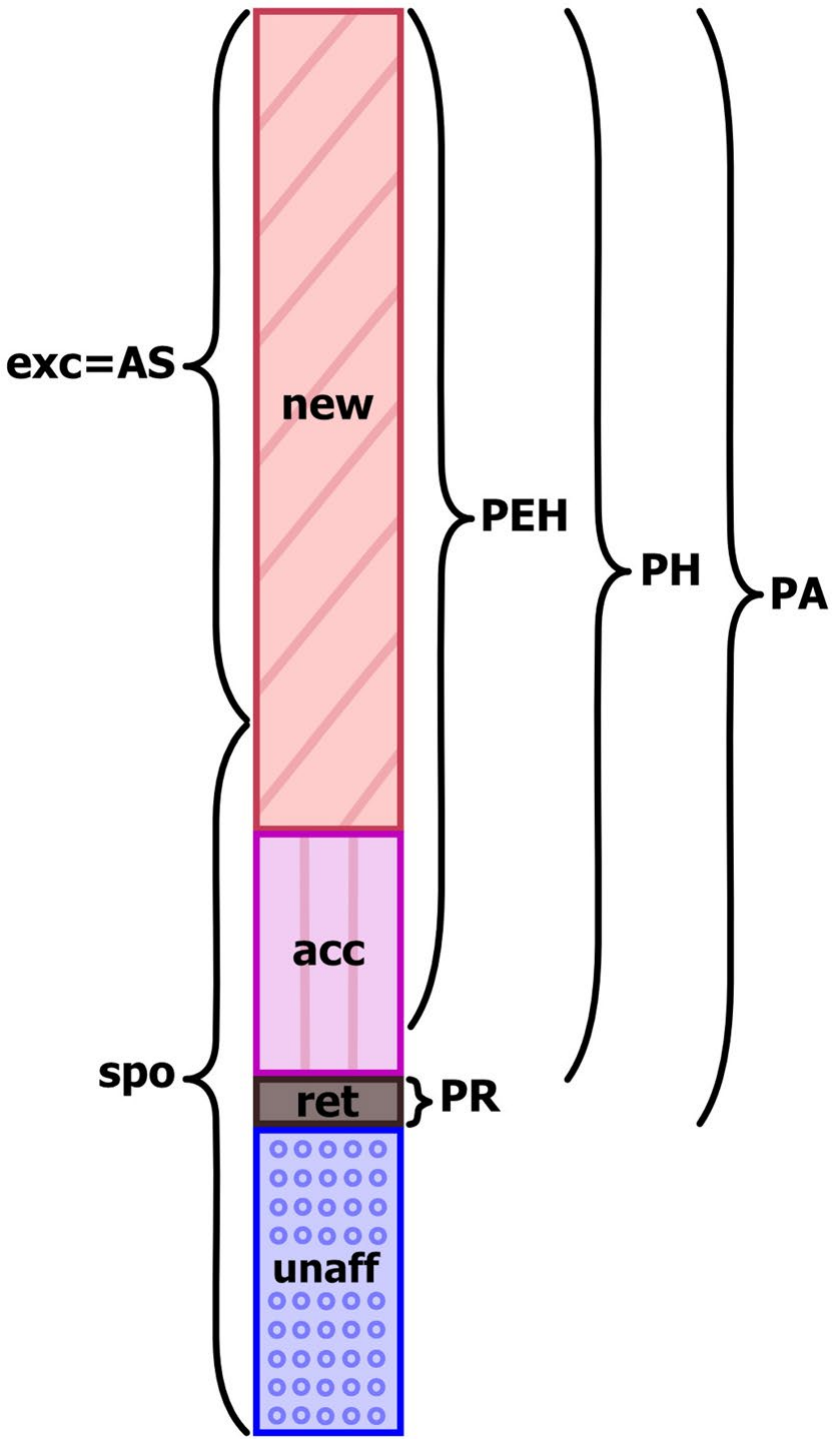
Graphical representation of fraction of cases and related cohort specific measures (*spo*  fraction of spontaneous cases, *exc* fraction of excess cases, *AS*  assigned share) and association measures (*PA*  probability of association, *PH*  probability of harm, *PEH*  probability of effective harm, *PR*  probability of retardation)

A graphical representation of these measures is shown in Fig. 6. They were calculated for all three mechanistic models and are further discussed below.

## Results

Table 2 presents the results of the three different models ${M}_{I}$, ${M}_{P}$ and ${M}_{P-ll}$ for the selected exposure scenario of 1 Gy at age 30 based on simulations of 1 million persons. The numbers show the mean and standard deviation for ten runs with different seed numbers of the random number generator. The uncertainties only represent the statistical uncertainties from the Monte Carlo sampling. The cancer cases were rounded back to integer numbers. The focus is on cancers in the age interval 60–61.

**Table 2:** Summary of simulation results

	Abbreviation/Definition	Model			
		${M}_{I}$ (SD)	${M}_{P}$ (SD)	${M}_{P-ll}$ (SD)	ERR
*Phenomenological level*					
Spontaneous cases	spo	695 (38)	654 (29)	711 (33)	568
Total cases	tot	1215 (33)	1277 (53)	1520 (29)	1363
Excess cases	exc = tot-spo	520 (23)	623 (38)	809 (45)	795
ERR	ERR = exc/spo	0.75 (0.07)	0.95 (0.06)	1.14 (0.11)	1.40
Assigned share (AS)	AS = exc/tot	0.43 (0.02)	0.49 (0.02)	0.53 (0.02)	0.58
*Mechanistic level*					
Cases unaffected by radiation	unaff	692 (38)	80 (9)	15 (6)	–
Acceleration (from 61–80 to 60–61)	acc	7 (3)	253 (21)	389 (17)	–
Retardation (from < 60 to 60–61)	ret	0 (0)	95 (6)	123 (10)	–
Radiation-induced new cases	new	516 (24)	849 (41)	993 (34)	–
*Measures of association*					
Probability of association (PA)	PA = (new + acc + ret)/tot	0.43 (0.02)	0.94 (0.006)	0.99 (0.004)	–
Probability of harm (PH)	PH = (new + acc)/tot	0.43 (0.02)	0.86 (0.005)	0.91 (0.007)	–
Probability of effective harm (PEH)	PEH = (new + acc-ret)/tot	0.43 (0.02)	0.79 (0.006)	0.83 (0.013)	–
Probability of retardation (PR)	PR = ret/tot	0 (0)	0.074 (0.003)	0.081 (0.007)	–
Harm / retardation ratio (HRR)	HRR = (new + acc)/ret	–	11.6 (0.4)	11.2 (1.0)	–

Numbers are given for the mean and standard deviation (SD) of 10 simulation runs with 1,000,000 persons. Cases in the age interval 60–61 years, in the absence of radiation (spontaneous cases), and after exposure of 1 Gy at age 30. At the mechanistic level, reflecting the biological mechanisms, it shows the number of cases that were observed in 60–61 after the exposure: cases where radiation had no effect, cases that were accelerated by radiation from older ages 61–80 to 60–61, cases that were retarded from younger ages to 60–61, and new cases, where the person would not be diagnosed with spontaneous cancer until age 80. Schematic representations of the contributions are also shown in Figs. 1 and 6. Different measures can be defined to quantify the association between cancer and radiation in the presence of acceleration and retardation

For the initiation model $M_{I}$, in the absence of radiation, 695 spontaneous cases in the 60–61 age interval were recorded. After exposure, the number of persons with cancer increased to a total of 1,215. Consequently, there are 520 excess cases with an ERR of 520/695 = 0.75, and the assigned share is AS = ERR/(1 + ERR) = 520/1215 = 0.43.

Individually, just 3 of the 695 persons with spontaneous cancer in the age interval 60–61 developed a cancer earlier in life due to radiation. In these three cases, radiation initiated a new clone that resulted in cancer before age 60. Similarly, seven persons who would have been diagnosed with spontaneous cancer between age 61 and 80, after exposure were diagnosed with cancer at age 60–61. For 516 persons, no spontaneous cancer would have occurred until age 80, but, with radiation, a cancer was diagnosed in the 60–61 age interval, so they are newly radiation-induced. There was no case retarded from age < 60 to the age group 60–61; therefore, the probability of association (PA), the probability of harm (PH) and the probability of effective harm (PEH) are identical. They are based on the sum of new and accelerated cases, PA = PH = PEH = (516 + 7)/1215 = 523/1215 = 0.43. For this model, PA, PH and PEH are very similar to AS since only three persons had cases that were additionally associated to radiation exposure. The uncertainties in the number of cases in the different categories are consistent with the underlying Poisson distribution.

In the model with an instantaneous effect on promotion, ${M}_{P}$, 654 spontaneous cancers were found in the age interval 60–61, and this number increased to 1,277 cases after the exposure. So, there are 623 excess cases resulting in an ERR of 0.95 and an assigned share of AS = 623/1277 = 0.49, a value similar to that produced by the ${M}_{I}$ model.

On an individual level, however, in the model ${M}_{P}$, many cancers were accelerated by radiation. Only 80 of the 654 spontaneous cancers were unaffected by the exposure and still appeared in the age interval 60–61. For 253 persons with a spontaneous cancer in the age interval 61–80, the cancers were accelerated by radiation to the age interval 60–61, and 849 persons without a spontaneous cancer until 80 are diagnosed with cancer in 60–61. In addition, for 95 persons, a spontaneous cancer before age 60 was retarded to the age interval 60–61 after exposure. The probability of association, based on the sum of new, accelerated and retarded cases, is given by PA = (849 + 253 + 95)/1277 = 1197/1277 = 0.94. The probability of harm from the new and accelerated cases is PH = (849 + 253)/1277 = 1102/1277 = 0.86. Subtracting the retarded cases results in a probability of effective harm PEH = (849 + 253–95)/1277 = 1007/1277 = 0.79. The probability that the observed cancer was retarded is given by the probability of retardation PR = 95/1277 = 0.074, and the harm/retardation ratio is HRR = PH/PR = (849 + 253)/95 = 11.6.

In the model ${M}_{P-ll}$ with lifelong promotion, 711 spontaneous cases, 1520 total cases and 809 excess cases were recorded. Thus, the ERR is 1.14 and the assigned share AS = 809/1520 = 0.53. Individually, only 15 spontaneous cases were unaffected by radiation, and also after radiation were recorded in the age interval 60–61. An acceleration from 61–80 to 60–61 was seen for 389 persons, and 993 persons without a spontaneous cancer were recorded with cancer in 60–61 after exposure. A retardation of cancer was found for 123 persons. Therefore, the probability of association PA = (993 + 389 + 123)/1520 = 1505/1520 = 0.99, the probability of harm PH = (993 + 389)/1520 = 1382/1520 = 0.91, and the probability of effective harm PEH = (993 + 389–123)/1520 = 1259/1520 = 0.83. The probability of retardation and the harm/retardation ratio are given by PR = 123/1520 = 0.081 and HRR = (993 + 389)/123 = 11.2. It is important to note that although the effects of stochasticity are larger in this model, i.e. 123 retarded cases, they are still small compared to the harmful effects that amount to 1,382 cases.

In comparison, the ERR model predicts a comparable number of excess and total cases. The predicted number of spontaneous cases is a bit lower than for the TSCE models, and thus the ERR, and consequently the AS, are somewhat higher. The differences in the ERR values between the models result mainly from different age dependencies. In the middle of the age interval 60–70, the risk values are closer, and the differences at age 60 reflect inherent model uncertainties (Kaiser et al. 2012). The ERR model does not describe biological mechanisms, and it is not possible to calculate the association measures.

## Discussion

The outcomes of three TSCE models with different biological mechanisms were investigated for an exposure to 1 Gy at age 30. Parameter values for all models were derived from the same breast cancer data set of the LSS. At the phenomenological level, the number of spontaneous baseline cases refers to the cancer cases expected in the absence of exposure to radiation, the total number of cases refers to all cancers observed in the presence of the studied exposure, while the excess cases are considered being produced by the studied radiation. As expected, the predicted numbers of spontaneous, radiation-induced excess and total cases were roughly similar among the models. For comparison, also a conventional ERR model was included with comparable number of cases. Consequently, also the assigned share AS was similar for all models with a range between 0.43 and 0.58.

To study detailed effects of radiation, a simulation was performed that tracked the development of cells towards cancer at the individual level, and assessed the influence of radiation on this process. The use of mechanistic biologically based models allows the identification of cancer cases that are not affected by radiation in any way, and separately of cancers that are accelerated or retarded. The number of unaffected cases (80 in the ${M}_{P}$ model) is smaller than the number of spontaneous cases (654). As shown by the simulation, the difference of 574 cases is composed of cancers that are accelerated by radiation out of the 60–61 age interval towards earlier ages (456 cases), cancers that are retarded towards the age interval 61–80 (83 cases), and persons for whom, after exposure, no cancer is observed any longer until age 80 (35 cases). The number of unaffected cases when added to the number of cases that are accelerated or retarded into the 60–61 age interval (253 cases and 95 cases, respectively) and the number of cases that are newly induced by radiation (849 cases) reproduce the total number of cases in the presence of the studied radiation (80 + 253 + 95 + 849 = 1,277 cases). These numbers of cases refer to the instantaneous promotion ${M}_{P}$ model, but the same logic applies to the other biological models.

Differences between the biological model results were found to be large. In particular, for the model with a radiation effect on initiation, all spontaneous cancers except three cases were unaffected by radiation, and only for seven persons, the cancer appeared at an earlier age after the exposure. On the other hand, for the two models with a radiation effect on promotion, the development was accelerated for many cases. A part of the cases that were observed in the 60–61 age interval after exposure would have spontaneously occurred until the end of follow-up, others might have occurred later or not at all and are designated as newly induced. In addition, cancers were also retarded from younger ages to the 60–61 age interval.

To quantify the radiation effect at the individual level, different measures of association were defined and investigated (Fig. 6). The probability of association, PA, represents the probability that the observed cancer has any association with radiation, being either newly induced, accelerated or retarded. The probability of harm PH is based only on the radiation-induced detrimental effects of new and accelerated cases. Likely, this is the quantity of most interest for an assessment whether the observed cancer is negatively associated to radiation. For the probability of effective harm PEH, the retarded cases are subtracted from the new and accelerated cases. While in the presence of radiation, both cancer acceleration and retardation can occur, and the predicted amount of these stochastic processes depends on the underlying model. By taking the difference between both quantities, the PEH is less dependent on stochastic fluctuations and might be more robust against different model assumptions. Additional measures to quantify the relevance of retardation are the probability of retardation PR and the harm retardation ratio HRR. In the absence of retardation effects, PA, PH and PEH are identical.

With a radiation effect on promotion, in a minority of cancer cases, a retardation effect was found, i.e. after radiation, the cancer appeared later than it would have been observed without exposure. In our study, it was assumed that the processes of cell division and differentiation/inactivation for all cells of the size-changed clone can be simulated independently from the cells of the original clone. This appears reasonable because with the radiation-induced size change also the microenvironment is modified, leading to a different local biological state (Fernandez-Antoran et al. 2019; Nakamura 2020) and, consequently, intercellular signalling will differ from the original clone. It is then possible that a clone that has increased in size by radiation will subsequently grow more slowly and become smaller than the original clone, and develops cancer at a later time; or a malignant event towards cancer might occur later in the larger clone instead of the smaller clone. The possibility of retardation is, therefore, an inevitable consequence of the assumed independence of cellular processes between the clones and the stochastic nature of cancer development. However, retardation is not an explicit radiation mechanism of the underlying mechanistic models. In principle, models with explicit retardation mechanisms are possible, e.g. models with decreased growth advantage of pre-cancerous cells due to radiation. To date, however, such models have not been shown to describe radioepidemiological data well.

The initiation model ${M}_{I}$ had only very few accelerated cases and no cases that were retarded. Therefore, in this case, AS is very similar to PA/PH/PEH. In the two promotion models, ${M}_{P}$ and ${M}_{P-ll}$, the large majority of cancers was shifted in age after exposure of 1 Gy. This resulted in values of PA of 0.94 and 0.99, respectively. The values for PH and PEH were in a range of 0.79–0.91. Therefore, in both models, the predicted probability that the observed cancer was negatively influenced by radiation was substantially higher than indicated by AS. The probability that the cancer was retarded by radiation was estimated to be 7.4% and 8.1%, respectively. Although the radiation effect on promotion acts differently in the two models—instantaneous versus lifelong—both models showed similar qualitative and quantitative behaviour. Overall, in the ${M}_{P-ll}$ model, more cancers were affected by radiation than in the ${M}_{P}$ model.

The definitions of the association measures always include the sum of the new and accelerated cases which is invariant against changes of follow-up time. For example, if the end of follow-up would be reduced to age 70 instead of age 80, the persons with a spontaneous cancer between 70 and 80 would not be counted any more as accelerated cases because their cancer would occur after the end of follow-up. However, they would be added to the new cases so that the sum of new and accelerated cases remains the same.

**Fig. 7:**
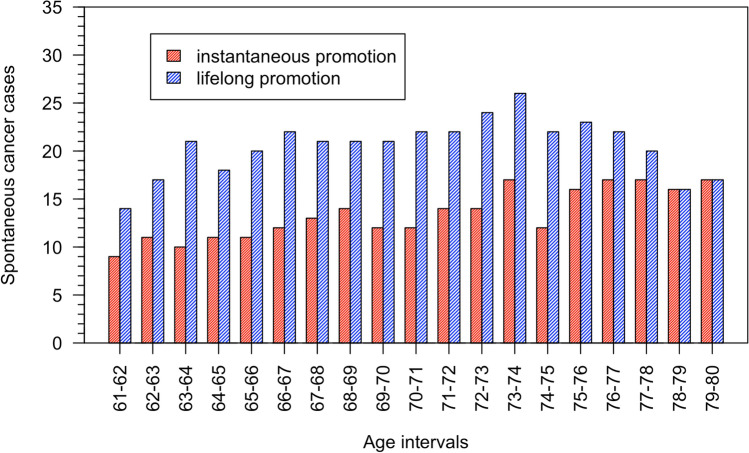
Age distribution of the spontaneous cancer cases that after exposure were accelerated to 60–61

Our study allows to calculate by how many years the cancers were accelerated according to the models. Figure 7 shows the age distribution of the spontaneous cancer cases that were accelerated by the exposure to the age interval 60–61 for the two promotion models. For example, for 12 persons in the model ${M}_{P}$ (22 persons in the model ${M}_{P-ll}$) with a spontaneous cancer between age 70 and 71 in the absence of exposure, the cancer was accelerated by the exposure by 10 years. The acceleration time is quite evenly distributed between 1 and 19 years. There is a slight tendency of the lifelong promotion model towards a larger fraction of accelerated cases at younger ages. For the 253 persons in the ${M}_{P}$ model, the average acceleration time was 10.9 years, and 10.2 years for the 389 persons of the ${M}_{P-ll}$ model.

While there is no doubt about the mutational effect of radiation, the potential role of radiation-induced clonal expansion is less clear and has become the focus of recent investigations. It was shown that doses as low as 50 mGy can lead to the preferential expansion of cells and clones carrying mutations in the Trp53 gene in mice (Fernandez-Antoran et al. 2019). From mice survival data, Nakamura (2020, 2021) inferred a tumour-promoting component of radiation that might be activated by induced changes to the microenvironment, e.g. by tissue inflammation. It was shown that effects of carcinogens on tumour promotion can generate actively growing lesions and accelerate clonal dynamics (Balmain 2020; Williams et al. 2022). Additional evidence stems from the analysis of radioepidemiological data with biologically based models. According to these studies, in almost all low-LET studies, a radiation action either on initiation, on clonal expansion, or both on initiation and clonal expansion was found. Furthermore, almost all high-LET studies for lung cancer after radon or plutonium inhalation showed dominance of a promotional mechanism (Rühm et al. 2017; UNSCEAR 2021).

The results of the simulation study depend on several assumptions. The end of follow-up was fixed at age 80 to allow for clearer interpretation. It is possible to remove this restriction and use instead real survival functions from population data. Each person can then be stochastically assigned an individual end of follow-up. While this leads to some shift in numbers, the effect on AS and the association measures is small (results not shown). The effect on AS and association measures for cancers diagnosed at attained ages other than the 60–61 age interval can, however, be further studied in more detail. A specific exposure scenario was chosen where the models are supported from the LSS data. Other scenarios with different ages and doses will modify the numbers, but not change the principal findings. Of particular interest would be analyses of values of the probabilities of association for multiple single (fractionated) or chronic exposures. In the simulation, it was assumed that the stochastic processes of cell division and differentiation are independent between a clone that was changed in size by irradiation and the original clone because of the differences in cellular communication. This seems a plausible assumption, but different biological mechanisms and implementations are possible and could be a focus of future research.

This work is focussed on developing concepts of associations, previously discussed by Greenland and collaborators (Beyea and Greenland 1999; Greenland 1999; Greenland and Robins 2000), using real epidemiological data. However, the presented results are still far from practical use. Only two-stage TSCE models with constant baseline parameters and without modifiers were used. An explicit age dependence of the baseline parameters, as present in Kaiser et al. (2012), was removed so that the cancer cases could be more transparently related to specific radiation mechanisms. Furthermore, other noxious agents such as chemicals could also contribute to carcinogenesis, and it might be difficult to separate different contributing factors. Most importantly, for any real application, a more profound knowledge on the biological mechanisms of radiation and how it affects cancer development is necessary. This can be achieved by future advanced integration of radiobiology, epidemiology and mechanistic modelling (UNSCEAR 2021). Here, we investigated only models with either a pure initiating or pure promotional effect of radiation. It is, however, likely that radiation has both an initiating and promoting component, though the relative importance of these contributions is unknown. It can be expected that models including both mechanisms result in values for cancer associations between the ones from models with only one of the two specific mechanisms.

Individual susceptibility to cancer was not taken into account in our study. There are indications that persons with BRCA mutations or a family history of breast cancer might be at higher risk not only for spontaneous but also for radiation-induced breast cancer (Bernstein et al. 2013; Eidemüller et al. 2021). This is an issue for future research as well.

To summarise, for a person who develops cancer after exposure, the individual association between cancer and radiation can be larger than AS, which is based solely on an assessment of the excess rate after the exposure. While the exact numbers depend on the models and assumptions, we believe that a robust result from this analysis is the dependence of the PA/PH/PEH on the underlying biological mechanisms. In a model with an effect of radiation on initiation, there is almost no earlier appearance of cancer, and AS approximates these association measures. However, if radiation acts on clonal expansion and promotes tumour development, many cancers could be shifted in age. The probability that the individual cancer is accelerated or newly induced is higher than expressed by AS. This general property does not seem to depend on the exact form of the radiation action, but only on the fact that radiation acts on promotion. Both of the two studied models, one with an instantaneous and one with a lifelong effect on promotion, predict values of PA/PH/PEH substantially higher than the AS.

Modern molecular methods are currently revolutionising our understanding of tumour development. Looking into the future, it might be possible to detect fingerprints of radiation in molecular data, and to identify relevant mechanisms. This will challenge the traditional view on cancer causation by radiation. How should an association be quantified if additional biological information is available? What is the role of a potential radiation-induced promotional mechanism? In this work, a first attempt was made to quantify the potential consequences of different biological mechanisms on individual association between cancer and radiation. Although the role of radiation as cancer-causing agent was the primary focus of this paper, the underlying principles are relevant for other carcinogens as well. Improved understanding of the effects of radiation on tumour development, and exploration of its impact on our view of causation and attributability will continue to be a challenging and fascinating topic of future research.

## Data Availability

LSS breast cancer data used in this analysis were made available by the Radiation Effects Research Foundation (RERF).
